# Emphysematous Pyelonephritis at 29 Weeks of Gestation: A Case Report and Review of the Literature

**DOI:** 10.7759/cureus.74943

**Published:** 2024-12-02

**Authors:** Hana Kijima, Yoshihiro Yoshimura, Daisuke Ishii, Kazumasa Matsumoto, Daigo Ochiai

**Affiliations:** 1 Obstetrics and Gynecology, Kitasato University School of Medicine, Sagamihara, JPN; 2 Urology, Kitasato University School of Medicine, Sagamihara, JPN

**Keywords:** emphysematous pyelonephritis, pregnancy, renal abscesses, type 2 diabetes mellitus, urinary tract infection

## Abstract

Emphysematous pyelonephritis (EPN) is a urinary tract infection progression characterized by gas retention in the renal tissues and a high mortality rate, but few cases have been reported. In this study, we present a 32-year-old primigravida with type 2 diabetes mellitus and a history of pyelonephritis who developed pyelonephritis at 29 weeks. Antimicrobial therapy was initiated; however, her clinical symptoms worsened. Ultrasonography and magnetic resonance imaging (MRI) led to a diagnosis of EPN. Fortunately, the patient improved with antibiotic therapy and delivered at 39 weeks.

Treatment for EPN may require invasive procedures such as percutaneous drainage or nephrectomy. Early diagnosis and timely intervention are crucial for the management of EPN during pregnancy. This case report highlights the need for imaging evaluation and tailored treatment strategies to balance maternal and fetal health in the treatment of EPN during pregnancy.

## Introduction

Urinary tract infections are a common perinatal complication, affecting approximately 8% of pregnancies [1], ranging from asymptomatic bacteriuria to pyelonephritis. Approximately 1-2% of pregnancies are complicated by bacterial pyelonephritis, with recurrence occurring in 20-25% of cases during pregnancy [1,2].

Emphysematous pyelonephritis (EPN) is a severe, acute necrotizing infection characterized by gas retention in the renal pelvis, renal parenchyma, and perirenal tissue, with a mortality rate of approximately 25% [3]. About 95% of EPN cases are associated with diabetes mellitus, and 25-75% with urinary tract obstruction [4,5]. Huang's severity classification determines treatment for EPN, and in addition to intravenous antibiotics, invasive procedures such as percutaneous drainage or nephrectomy may be required in severe cases [3,5]. However, there are few reports of EPN during pregnancy, and treatment strategies are not well-defined. Here, we report a case of EPN at 29 weeks of pregnancy.

## Case presentation

A 32-year-old primigravida woman was referred to our hospital at nine weeks of gestation with complications including type 2 diabetes mellitus, a history of pyelonephritis before pregnancy, and obesity (non-pregnant body mass index: 35 kg/m^2^). She had been treated with anti-diabetic medications (metformin hydrochloride 500 mg/day and dapagliflozin propylene glycolate hydrate 10 mg/day) before her pregnancy. When she became pregnant, she stopped her diabetic medications and switched to insulin therapy.

The patient was initiated on insulin detemir at a dose of 7 units/day and insulin aspart at 38 units/day at eight weeks of gestation. Despite this regimen, glycemic control remained suboptimal, necessitating an increase in the doses to insulin detemir 49 units/day and insulin aspart 171 units/day by 30 weeks of gestation. However, due to persistent hyperglycemia, dietary therapy was intensified, resulting in a reduction of insulin requirements to 4 units/day of insulin detemir and 87 units/day of insulin aspart by 38 weeks of gestation. Glycemic control, as evaluated by hemoglobin A1c (HbA1c) levels, improved progressively throughout pregnancy, with values recorded at 6.6%, 6.7%, and 6.2% at seven, 30, and 38 weeks of gestation, respectively.

**Figure 1:**
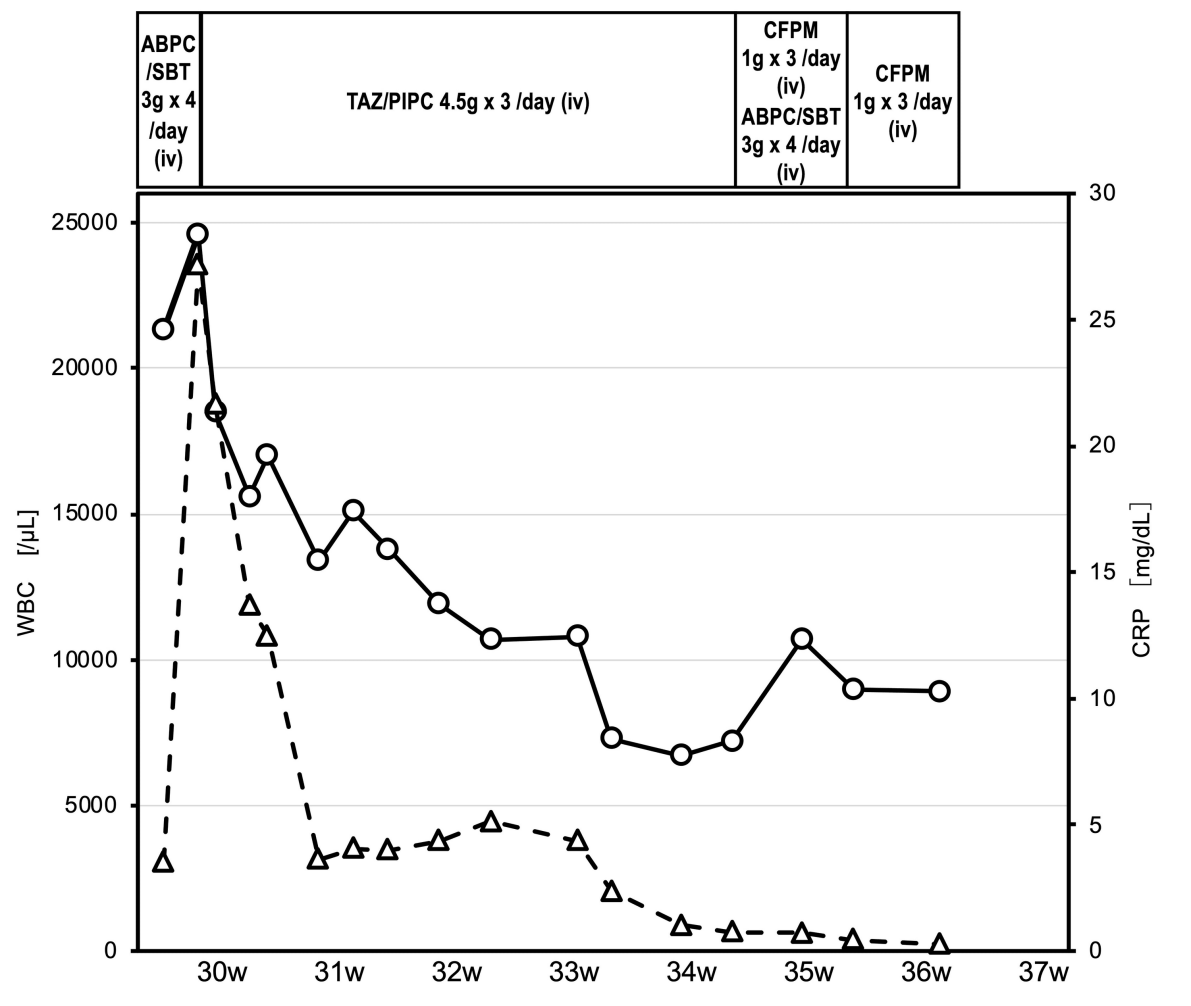
Clinical course of the patient Antimicrobial therapy with ampicillin/sulbactam was initiated; however, the patient’s clinical symptoms worsened. This switch to tazobactam/piperacillin was highly effective. The antibiotic therapy was continued until 36 weeks gestation. The solid line represents white blood cell count and the dashed line represents C-reactive protein. ABPC/SBT: ampicillin/sulbactam; TAZ/PIPC: tazobactam/piperacillin; CFPM: cefepime; iv: intravenous; WBC: white blood cells; CRP: C-reactive protein

Her pregnancy was uneventful until she experienced right flank pain and fever at 29 weeks and four days of gestation. Her vital signs included a fever of 39.0℃, blood pressure of 140/80 mmHg, heart rate of 115 bpm, and respiratory rate of 18 breaths/min. There were no signs of intrauterine infection, and intravenous antimicrobial treatment with sulbactam/ampicillin was initiated (Figure 1). However, her condition worsened after two days, with elevated inflammatory markers: white blood cells (26,300 cells/µL) and serum C-reactive protein (27.15 mg/dL).

**Figure 2:**
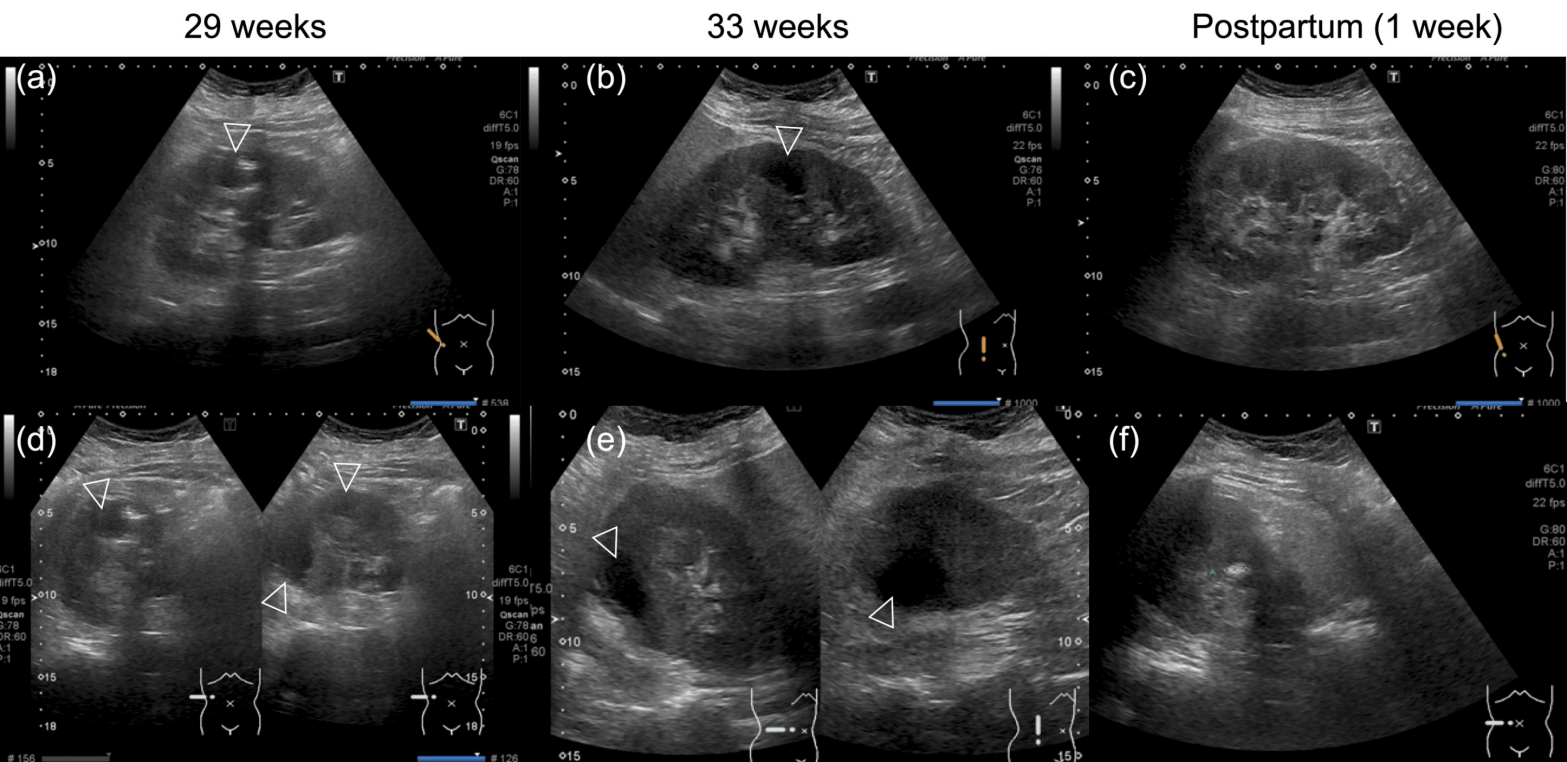
Ultrasound images of emphysematous pyelonephritis Heterogeneous mass mixed with hyperintense and hypointense areas on long-axis (a-c) and short-axis (d-f) ultrasound images. Ultrasonography confirmed renal abscesses (arrowheads) at 29 weeks of gestation (a,d). The abscess was also detected at 33 weeks of gestation (b,e) but gradually shrank and resolved after birth (c,f).

**Figure 3:**
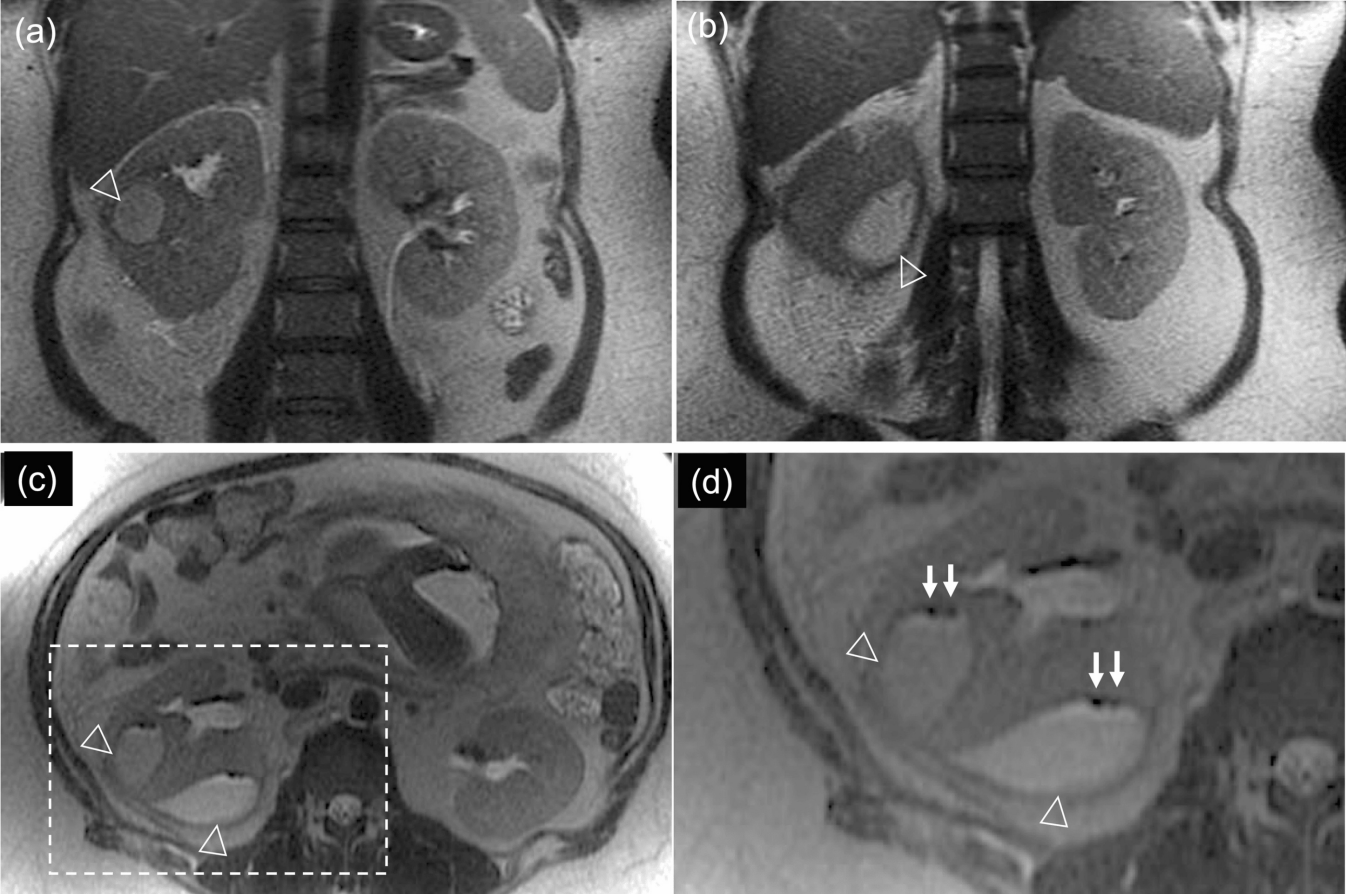
T2-weighted magnetic resonance images at 30 weeks of gestation Multiple renal abscesses (arrows) in the right kidney showed hyperintensity in the coronal (a,b) and transverse (c,d: magnified view of the dotted square area) views, while air within the abscesses (arrowheads) appeared hypointense (d).

Ultrasonography of the right kidney showed a heterogeneous mass with hyperintense and hypointense areas (Figure 2), and T2-weighted magnetic resonance imaging (MRI) at 30 weeks of gestation revealed multiple air-containing abscesses (Figure 3), leading to a diagnosis of EPN with a class II Huang severity classification. The urine culture detected *Escherichia coli*, but the blood culture test proved negative. The antibiotics were changed to tazobactam/piperacillin (Figure 1); if this antimicrobial treatment did not improve her condition within three days, we planned to deliver the fetus and perform percutaneous drainage.

She was hospitalized for 52 days, from 29 weeks four days to 36 weeks six days gestation, and received antibiotics treatment. Fortunately, her clinical symptoms and inflammation improved following the switch to tazobactam/piperacillin and the abscess gradually shrunk (Figures 1-2). The patient subsequently developed a hypertensive disorder during pregnancy. At 39 weeks, labor induction was attempted; however, a cesarean section was performed because of labor arrest. The neonate, a boy weighing 3,382 g, was born with Apgar scores of eight and nine at 1 min and 5 min, respectively. Seven days after the cesarean section, the patient was discharged without complications, and an ultrasound examination at one week post-cesarean confirmed the resolution of the renal abscess (Figure 2).

## Discussion

In the present case, our patient developed EPN caused by *E. coli* at 29 weeks gestation following an episode of recurrent pyelonephritis. Antibiotic treatment with piperacillin/tazobactam was highly effective, leading to favorable outcomes for both the mother and infant. EPN during pregnancy is a rare occurrence; thus, few cases have been reported and there is no consensus regarding optimal treatment.

Diabetes mellitus and urinary tract stasis during pregnancy may have contributed to the development of EPN in this case. The causative organisms are typically *Enterobacteriaceae*gram-negative rods, mainly *E. coli *and *Klebsiella* spp. [4], which produce acids and gases via anaerobic metabolism under hypoxic conditions caused by high sugar concentrations in tissues and chronic blood flow obstruction [3-5]. Moreover, it has been suggested that hyperglycemia may decrease cytokine production, such as IL-2, IL-6, and IL-10, impair leukocyte function, and impair leukocyte defense against infection. Some reports suggest that impaired glucose tolerance increases urosepsis [6], while others suggest that high HbA1c is not an independent risk factor for urinary tract infection [7], indicating that the association between glycemic control and EPN is controversial. Although EPN was found to predominantly affect the left kidney in non-pregnant patients [3-5], the right kidney lesion in our case may be attributed to the anatomy of the gestational uterus, which can cause right ureteral compression and urinary tract stasis after the second trimester. 

Early diagnosis, staging, and treatment are critical for the management of EPN [3,5]. Abdominal radiography or renal ultrasound is recommended for diabetic patients with pyelonephritis, followed by computed tomography (CT) in non-pregnant states [3,5]. Huang's classification system categorizes EPN severity [3], and in our case, MRI was used to avoid radiation exposure during pregnancy, confirming a class II diagnosis. Since the diagnostic accuracy of EPN by ultrasound alone is only approximately 65% [8], additional evaluation with MRI or CT is essential for accurate diagnosis and appropriate management of EPN in pregnant patients with refractory pyelonephritis.

In non-pregnant patients, antimicrobial treatment combined with percutaneous drainage is recommended in cases of EPN [5]. Ultrasound evaluation of treatment efficacy is recommended approximately every three days, and a more aggressive invasive procedure such as nephrectomy is recommended if the abscess persists [5]. However, in pregnant patients, it is difficult to perform percutaneous drainage for all abscesses owing to postural position limitations, such as the inability to lie prone. Physiological changes during pregnancy also make it difficult to drain pus, and in some cases, frequent exchanges worsen the infection [9]. Moreover, CT-guided drainage cannot be performed due to concerns regarding radiation exposure during pregnancy. Previous reports indicated that 75-95% of patients experienced clinical improvement within 2-3 days of the initiation of intravenous antibiotics for acute pyelonephritis [1]. However, since several cases have been reported showing rapid exacerbation of the disease within a few days after the onset, leading to a poor prognosis for mother and child [10,11], it is recommended to evaluate the therapeutic efficacy every three days during pregnancy [3]. We, therefore, planned to deliver the fetus and then proceed to invasive treatment if the patient did not exhibit apparent clinical improvement within three days after initiating the piperacillin/tazobactam treatment. The patient was treated with antibiotics based on the Japanese "Platinum Manual of Infectious Diseases 2020." The manual does not define the duration of antibiotic therapy for EPN but recommends that antibiotics be administered until the abscess disappears on imaging studies in renal abscesses. Therefore, the antibiotics treatment was not finished at 34 weeks of gestation when C-reactive protein was normalized because the renal abscess remained on ultrasound examination, and the treatment was continued until 36 weeks of gestation.

To date, only five cases of EPN during the perinatal period, including our case, have been reported (Table 1) [12-15]. Of these, three occurred during pregnancy and two occurred in the postpartum period. The cases were characterized by a history of pyelonephritis in three patients, kidney stones in two, and diabetes mellitus in two. In one postpartum case, pyelonephritis initially manifested during pregnancy and progressed to EPN after delivery. The other postpartum patient developed EPN shortly after childbirth, suggesting an antepartum onset of infection. In these perinatal patients, EPN was found to affect both kidneys at nearly equal frequencies. In the two cases involving the left kidney, a history of left-sided kidney stones was noted, potentially increasing the susceptibility to EPN on that side during the perinatal period. All patients were administered antimicrobial therapy. However, in one case, intrauterine fetal death occurred immediately after antibiotic administration [13]. Percutaneous drainage was performed in two cases: one at six weeks of gestation [12] and another at four days postpartum [14]. Percutaneous nephrolithotomy was performed during the postpartum period [15].

**Table 1:** Reported cases of emphysematous pyelonephritis during the perinatal period GW: gestational weeks; PP: postpartum; ND: not determined

Case	Age	GW	Medical history	Class	Side	Treatment	Causative organisms	Maternal outcome	Fetal outcome
Gaither et al. [12]	37	6w	Pyelonephritis, kidney stone	ⅢB	Left	Antibiotic, percutaneous drainage	*Citrobacter freundii*, *Pseudomonas aeruginosa*	Favorable	ND
Grozel et al. [13]	26	16w	Diabetes mellitus	Ⅳ	Bilateral	Antibiotic	Escherichia coli	Favorable	Fetal death
Our case	32	29w	Pyelonephritis, diabetes mellitus	Ⅱ	Right	Antibiotic	Escherichia coli	Favorable	Favorable
Kumar et al. [14]	29	PP4day	None	Ⅳ	Bilateral	Antibiotic, percutaneous drainage	*Escherichia coli*, *Klebsiella*	Favorable	Favorable
Hammad et al. [15]	30	PP3w	Pyelonephritis, kidney stone	Ⅱ	Left	Antibiotic, percutaneous nephrolithotomy	Escherichia coli	Favorable	ND

EPN is rare in perinatal patients and has an extremely poor prognosis in cases in which appropriate treatment is not provided. Reports of percutaneous drainage for renal abscesses during pregnancy are limited, and several cases were associated with poor obstetric outcomes such as intrauterine fetal death [10,11] and preterm delivery [16]. Thus, percutaneous drainage of renal abscesses during pregnancy should be considered a high-risk procedure. In the case of EPN during pregnancy, immediate delivery followed by invasive treatment should be considered in addition to the treatment while continuing the pregnancy.

## Conclusions

In conclusion, although urinary tract infections are common during pregnancy, some patients experience acute exacerbations. Further imaging evaluation using ultrasound combined with MRI or CT should be performed immediately for such patients. The timing of invasive intervention and delivery should be based on the efficacy of antimicrobial therapy, percutaneous drainage risk, and infant prematurity in pregnant patients with EPN. Fortunately, in our case, the patient's condition improved with antibiotic therapy alone.
